# Longevity in response to lowered insulin signaling requires glycine N‐methyltransferase‐dependent spermidine production

**DOI:** 10.1111/acel.13043

**Published:** 2019-11-13

**Authors:** Luke S. Tain, Chirag Jain, Tobias Nespital, Jenny Froehlich, Yvonne Hinze, Sebastian Grönke, Linda Partridge

**Affiliations:** ^1^ Max‐Planck Institute for Biology of Ageing Cologne Germany; ^2^ Institute of Healthy Ageing, and GEE UCL London UK

**Keywords:** aging, autophagy, IGF, insulin, lifespan, metabolism, polyamine

## Abstract

Reduced insulin/IGF signaling (IIS) extends lifespan in multiple organisms. Different processes in different tissues mediate this lifespan extension, with a set of interplays that remain unclear. We here show that, in *Drosophila,* reduced IIS activity modulates methionine metabolism, through tissue‐specific regulation of glycine *N*‐methyltransferase (Gnmt), and that this regulation is required for full IIS‐mediated longevity. Furthermore, fat body‐specific expression of *Gnmt* was sufficient to extend lifespan. Targeted metabolomics showed that reducing IIS activity led to a Gnmt‐dependent increase in spermidine levels. We also show that both spermidine treatment and reduced IIS activity are sufficient to extend the lifespan of *Drosophila,* but only in the presence of Gnmt. This extension of lifespan was associated with increased levels of autophagy. Finally, we found that increased expression of Gnmt occurs in the liver of liver‐specific IRS1 KO mice and is thus an evolutionarily conserved response to reduced IIS. The discovery of Gnmt and spermidine as tissue‐specific modulators of IIS‐mediated longevity may aid in developing future therapeutic treatments to ameliorate aging and prevent disease.

## Graphical abstract

Increased tissue‐specific expression of Glycine N‐methyltransferase (Gnmt), a central enzyme in the methionine cycle, and the polyamine spermidine modulate lifespan through autophagy in response to reduced insulin/IGF signaling activity in *Drosophila*. Increased expression of Gnmt in response to reduced IIS activity is conserved in mammals making Gnmt and spermidine possible future therapeutic targets to ameliorate the effects of ageing.

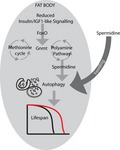

## 1 INTRODUCTION

Aging is the primary risk factor for human cardiovascular disease, diabetes, cancer, and neurodegenerative disorders including Alzheimer's disease (Johnson, Dong, Vijg, & Suh, 2015; Partridge, Deelen, & Slagboom 2018). Mechanisms of aging can be modulated in model organisms, extending both lifespan and healthspan through genetic, environmental, and pharmacological interventions (Fontana, Partridge, & Longo, 2010; Kenyon, 2010; Longo et al., 2015). Importantly, these lifespan‐extending perturbations function through mechanisms that are highly conserved in evolution (Fontana et al., 2010). Aging is thus no longer viewed as an inevitable process of age‐related decline.

Studies examining the transcriptomic and proteomic changes that occur during aging, or in response to interventions that ameliorate its effects, have revealed several conserved prolongevity processes, including many metabolic changes (Afschar et al., 2016; Dobson et al., 2018; Hahn et al., 2017; Murphy et al., 2003; Narayan et al., 2016; Page et al., 2018; Stout et al., 2013; Tain et al., 2017; Teleman, Hietakangas, Sayadian, & Cohen, 2008). These include carbohydrate metabolism (Afschar et al., 2016), lipid and fatty acid metabolism (Dobson et al., 2018; Hahn et al., 2017; Murphy et al., 2003; Page et al., 2018), energy metabolism (Afschar et al., 2016), and protein and methionine (Met) metabolism (Narayan et al., 2016; Stout et al., 2013).

Met metabolism is closely linked to aging in diverse model organisms (McIsaac, Lewis, Gibney, & Buffenstein, 2016). Restricting the availability of dietary Met is sufficient to increase lifespan in mice and flies (Grandison, Piper, & Partridge, 2009; Miller et al., 2005). However, the role of Met in determination of longevity is complex (McIsaac et al., 2016). Met is an essential amino acid, encoded for by the initiation codon, and is thus the first amino acid in newly formed polypeptides during conventional translation. The role of Met in protein translation, in the creation of other amino acids (e.g., cysteine), and in producing metabolites such as S‐adenosyl methionine (SAM) or glutathione makes the metabolism of Met vital for cellular and organismal health. At the core of the Met metabolism cycle, Met is converted to SAM, which in turn is converted into S‐adenosyl homocysteine (SAH), and finally into homocysteine. From the main core of Met metabolism, metabolites can also branch off into distinct metabolic pathways. For example, homocysteine is an essential metabolite in the production of cysteine within the transsulfuration pathway (TSP) (Kabil & Banerjee, 2010). Downstream of the TSP, cysteine is also essential for the production of the antioxidant glutathione. SAM is a versatile metabolite of Met that acts as a major cellular methyl donor and thus plays an important role in several cellular processes including epigenetic regulation, protein modification, and lipid metabolism (Lu & Mato, 2012). SAM can also branch from the Met cycle and enter into the polyamine synthesis pathway, resulting in the production of spermidine and other polyamine substrates (Pegg, 2009). Interestingly, changes in SAM metabolism (Obata & Miura, 2015), TSP activity (Hine et al., 2015; Kabil, Kabil, Banerjee, Harshman, & Pletcher, 2011), glutathione metabolism (Ayyadevara et al., 2005), and spermidine treatment (Eisenberg et al., 2016, 2009), like reductions in IIS activity, can all extend lifespan in a variety of model organisms. However, whether changes in Met metabolism and its associated pathways underlie the extensions is unclear.

Glycine N‐methyl transferase Gnmt is a central enzyme within Met metabolism, catalyzing the transfer of a methyl group from SAM to glycine to form sarcosine and SAH (Luka, Mudd, & Wagner, 2009), acting downstream of IIS (Obata & Miura, 2015). We have identified an evolutionarily conserved, and tissue‐specific, role for Gnmt and polyamine biosynthesis in lifespan determination, downstream of reduced IIS in *Drosophila*. *Gnmt* is required for IIS‐mediated longevity, while fat body‐specific expression of Gnmt is sufficient to extend longevity in flies. The prolongevity polyamine spermidine is increased in IIS mutants and is required for *Gnmt*‐dependent, IIS‐mediated longevity. Furthermore, spermidine‐mediated and Gnmt‐mediated longevity occur through increased autophagy. Finally, we show that this response to reduced IIS is conserved in the liver of liver‐specific IRS1 KO mice, suggesting that modulating Gnmt activity, and/or the synthesis of spermidine, represents possible targets for anti‐aging therapeutics.

## 2 RESULTS

### 2.1 Reduced IIS increases expression of Gnmt in the fat body

**Figure 1:**
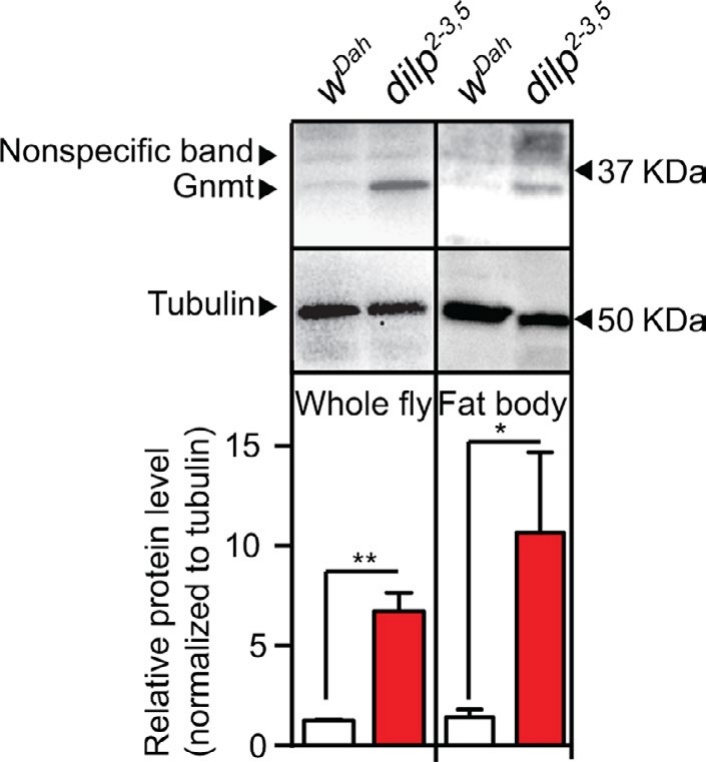
Increase in expression of Gnmt in response to reduced IIS. Western blot analysis of whole fly (*n* = 3) or fat body‐specific (*n* = 6) *Gnmt* protein levels from *w^Dah^* (control) and *dilp^2‐3,5^* mutant flies (significance determined by *t* test *p*‐value *< .05, **< .01)

In mammals, Gnmt is a key regulator of the Met cycle and is mostly expressed in the liver (Uhlén et al., 2015). In mice, expression of Gnmt declines with age (Armstrong, Rakoczy, Rojanathammanee, & Brown‐Borg, 2014). To determine the tissue‐specific expression of Gnmt in *Drosophila* and its response to reduced IIS, we performed Western blot analysis to quantify Gnmt protein expression in flies lacking 3 of the 7 insulin‐like peptides, *dilp2‐3,5* mutant flies (Grönke, Clarke, Broughton, Andrews, & Partridge, 2010). In control flies (*w^Dah^*), Gnmt protein was detected in total fly and fat body protein extracts (Figure 1), but not in the thorax or the gut (Figure S1). Gnmt levels were substantially increased in the whole fly and the fat body of *dilp2‐3,5* mutant flies (Figure 1), but again were not detected in the thorax or the gut (Figure S1). This suggests that increased expression of Gnmt in the fat body may play a role in modulating the phenotypes induced by reduced IIS, possibly including longevity.

### 2.2 Ectopic expression of Gnmt in the fat body extends lifespan

**Figure 2:**
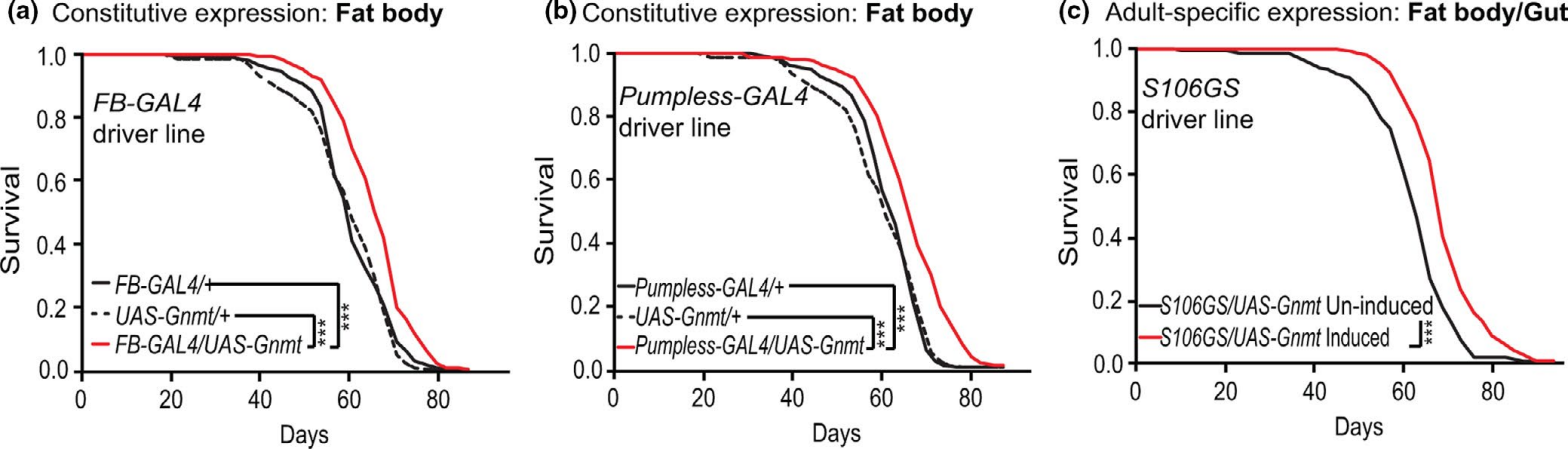
Fat body‐specific ectopic expression of *Gnmt* extends lifespan. Survival analysis of flies ectopically expressing UAS‐*Gnmt* driven by two independent constitutive fat body GAL4 driver lines (a) *FB‐GAL4* (*FB‐GAL4/UAS‐Gnmt*), (b) *Pumpless‐GAL4* (*pumpless‐GAL4/UAS‐Gnmt*), and (c) adult‐specific over‐expression of *UAS‐Gnmt* using the GeneSwitch driver *S106* induced by RU486 (200 μM) compared to uninduced controls (ETOH) (*n* = 150 flies per condition, *p* < .001, log‐rank test). Lifespan analyses of genetic controls are shown in Figure S2b

To determine whether increasing the level of Gnmt in the fat body was sufficient to extend lifespan, we over‐expressed Gnmt using a *UAS‐Gnmt* construct (Obata & Miura, 2015) and two independent, constitutive, fat body drivers, *Fat Body‐Gal4* (*FB‐Gal4*) (Grönke et al.,^2003^) and *Pumpless‐Gal4* (Zinke, Kirchner, Chao, Tetzlaff, & Pankratz, 1999). Both *FB‐Gal4* and *Pumpless‐Gal4* drivers led to increased *Gnmt* transcript levels in the fat body (Figure S2a,b) and extended lifespan, by 10% and 8%, respectively, compared to controls (Figure 2a,b). To prevent possible developmental effects of *Gnmt* expression, *UAS‐Gnmt* was then expressed using the adult‐specific *S106GS* GeneSwitch driver line (Osterwalder, Yoon, White, & Keshishian, 2001), which is expressed in fat body and gut. Inducing expression of *UAS‐Gnmt* with the *S106GS* driver increased *Gnmt* levels ~ 3‐fold in the fat body compared to uninduced controls (Figure S2d) and significantly extended lifespan by 9% (Figure 2c, Figure S2e). To determine whether the over‐expression of Gnmt in the gut played a role in the extension of lifespan, we drove expression only in the gut using the constitutive, gut‐specific driver *NP1‐Gal4* (Jiang et al., 2009)*.* Using *NP1‐Gal4*, the gut‐specific expression of *Gnmt* was comparable to that of fat body‐specific over‐expression (Figure S2c); however, lifespan in these flies was unchanged (Figure S2f). These findings indicate that increased Gnmt protein, specifically in the fat body, can extend lifespan.

### 2.3 Gnmt is necessary for full extension of lifespan by lowered IIS

**Figure 3:**
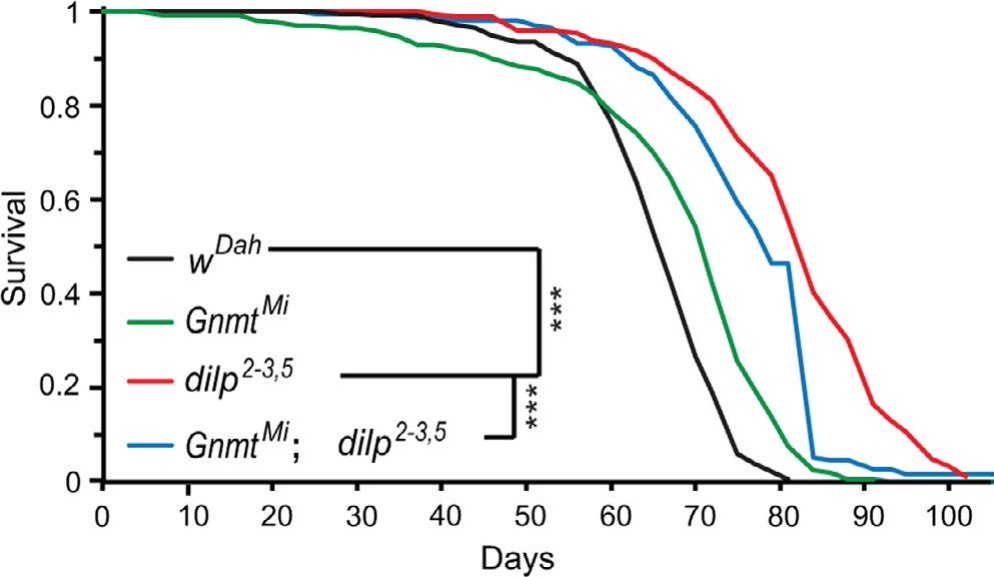
Gnmt is required for IIS‐mediated longevity. Survival analysis of *w^Dah^* control (black), *Gnmt^Mi^* mutant (green), *dilp2‐3,5* mutant (red), and *Gnmt^Mi^;dilp2‐3,5* (blue) double mutants (*n* = 150 flies per condition, *p* < .001)

To determine whether Gnmt is required for increased lifespan in response to lowered IIS, we measured lifespan of the long‐lived *dilp2‐3,5* mutants (Grönke et al., 2010) in the presence and absence of *Gnmt*. Loss of *Gnmt* (*Gnmt^Mi04290^,* hereafter referred to as *Gnmt^Mi^*) did not reduce lifespan of wild‐type flies (Figure 3), indicating that Gnmt is not essential for normal lifespan. However, the extended lifespan of *dilp2‐3,5* mutants was significantly diminished in the absence of *Gnmt* (*Gnmt^Mi^,dilp2‐3,5*), with the double mutants living 33% less long than the *dilp2‐3,5* mutants alone (Figure 3). Gnmt is thus necessary for the full extension of lifespan of *dilp2‐3,5* mutants.

### 2.4 Gnmt enhances longevity independently of resistance to either oxidative or xenobiotic stress, and the transsulfuration pathway

**Figure 4:**
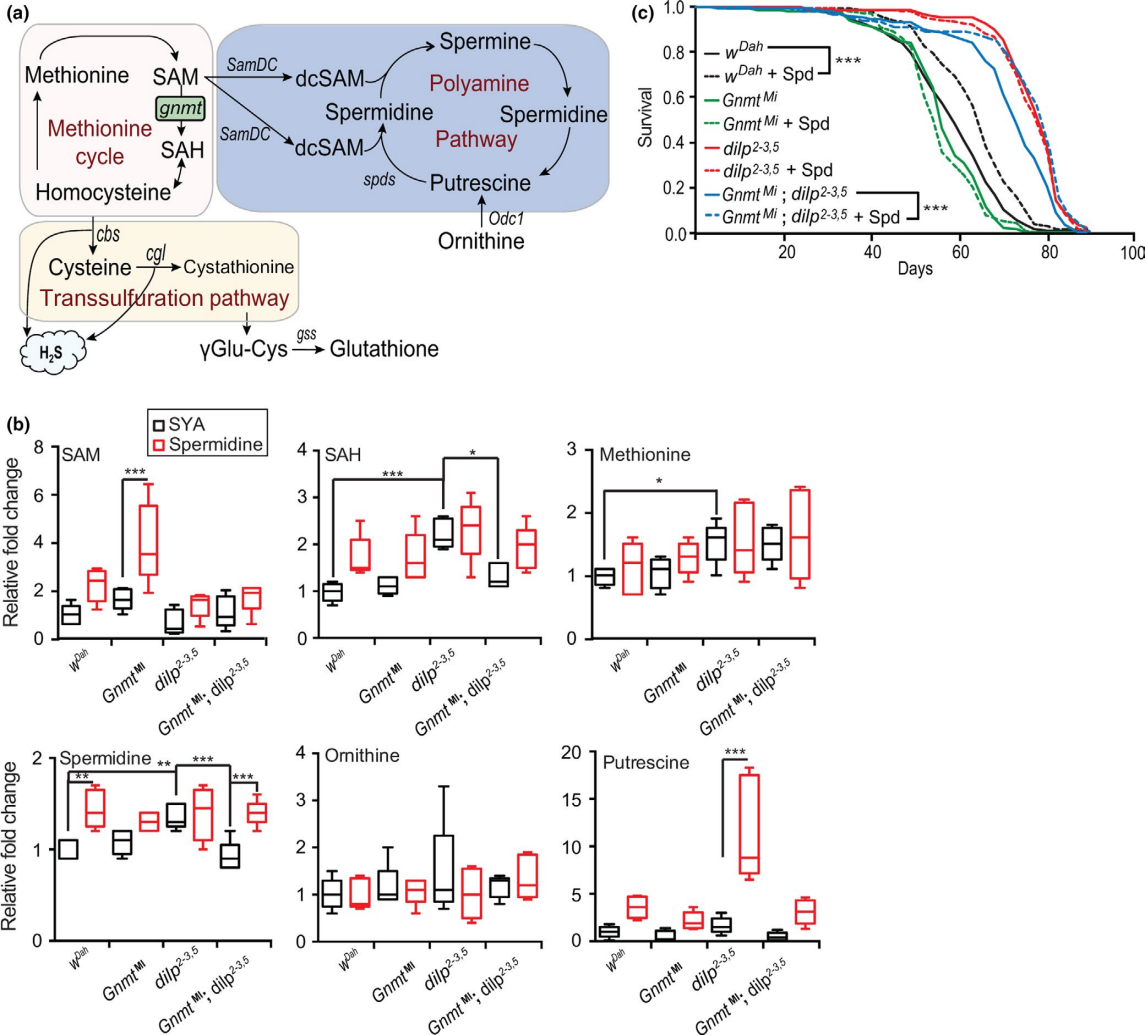
Gnmt influences longevity via polyamine pathway. (a) Methionine, TSP, glutathione, and polyamine pathway schematic. (b) UPLC‐MS/MS analysis of SAM, SAH, methionine, spermidine, putrescine, and ornithine in 10‐d‐old female *w^Dah^, Gnmt^Mi^, dilp2‐3,5*, and *Gnmt^Mi^;dilp2‐3,5* flies in spermidine‐treated and untreated (SYA) conditions (*n* = 5, significance determined by one‐way ANOVA for control fed comparisons, and by 2‐way ANOVA for genotype and spermidine treatment interaction tests, *p*‐value *< .05, **< .01, ***< .001). TSP and glutathione metabolite quantification are shown in Figure S4b. (c) Survival analysis of wild‐type *w^Dah^*, *Gnmt^Mi^* mutant, *dilp2‐3,5*, and *Gnmt^Mi^;dilp2‐3,5* double mutants fed control (SYA) food or fed 1mM of spermidine (Spd) (*n* = 150 flies per condition, *p* < .001, log‐rank test)

Gnmt is a key enzyme in methionine metabolism, catabolizing SAM into SAH. To determine the molecular mechanism underlying the prolongevity role of Gnmt in response to lowered IIS, we focused on Met and its associated metabolic pathways. These include the transsulfuration pathway (TSP), oxidative and xenobiotic detoxification pathway (glutathione metabolism), and the polyamine pathway (Lu & Mato, 2012; Minois, Carmona‐Gutierrez, & Madeo, 2011) (Figure 4a).

Ubiquitous, or fat body‐specific, over‐expression of Gnmt increases SAM catabolism (Obata & Miura, 2015), possibly converting it into sarcosine and S‐adenosyl homocysteine (SAH). SAH can then be hydrolyzed to homocysteine by SAH hydrolase. Homocysteine is, in turn, the primary source of cysteine for the TSP and the downstream production of glutathione (Kabil et al., 2011). Therefore, increasing Gnmt expression may elevate SAH, cysteine, and glutathione levels and protect against oxidative stress. To test this idea, we investigated whether over‐expression of GNMT in the fat body was sufficient to confer resistance to oxidative stress, induced by hydrogen peroxide (H_2_O_2_). We found no change in the survival of flies over‐expressing Gnmt using *FBGal4, pumplessGal4, or S106GS* drivers, suggesting that resistance to oxidative stress is not causal for the longevity of these flies (Figure S3a,b). This is concordant with the finding that oxidative stress resistance may not be causal for the longevity of IIS mutants (Afschar et al., 2016; Slack, Giannakou, Foley, Goss, & Partridge, 2011). Furthermore, *S106GS > UAS‐Gnmt* flies did not show increased resistance to the xenobiotic dichlorodiphenyltrichloroethane (DDT), suggesting that resistance to xenobiotics is also not causal for lifespan extension in these flies (Figure S3c).

TSP activity leads to the release of hydrogen sulfide (H_2_S) gas (Figure 4a), exposure to which can increase the lifespan of *C. elegans* (Miller & Roth, 2007). More recently, H_2_S and TSP activity have been shown to correlate with the effects of dietary restriction, including extension of lifespan, in yeast, worm, fruit fly, and rodent models (Hine et al., 2015). The TSP could, therefore, mediate longevity of *Gnmt*‐expressing flies, through H_2_S production. To test this idea, we analyzed H_2_S production capacity as a readout of TSP activity, but could not detect any change in H_2_S levels in *FBGal4 > UAS‐Gnmt, pumplessGal4 > UAS‐Gnmt*, or *S106GS > UAS‐Gnmt* flies (Figure S3d,e). Together, these findings suggest that neither the TSP nor glutathione production branches of Met metabolism underlie the longevity of *Gnmt*‐over‐expressing flies.

### 2.5 Gnmt promotes longevity via the polyamine synthesis pathway

Met metabolism also branches, through SAM, into the polyamine pathway (Pegg, 2009) (Figure 4a). SAM is decarboxylated by SAM decarboxylase (*SamDC*) and combines with putrescine to form spermidine, or with spermidine to form spermine via spermidine synthase (SpdS) (Pegg, 2009). In rodents and humans, spermidine levels decrease with age (Das & Kanungo, 1982; Vivó et al., 2001). Furthermore, dietary supplementation with spermidine is sufficient to extend the longevity of yeast, worms, flies, and mice (Eisenberg et al., 2016, 2009). If changes in the polyamine pathway underlie IIS‐mediated lifespan extension, we would expect either the expression or the activity of polyamine synthesis enzymes to be elevated in *dilp2‐3,5* mutants. However, expression of the two rate‐limiting enzymes in polyamine synthesis, *SamDC* and *Odc1,* was not changed in *dilp2‐3,5* mutant flies (Figure S4a).

To determine whether polyamine synthesis enzyme activity underlies Gnmt‐mediated *dilp2‐3,5* mutant longevity, we quantified key metabolites of the methionine cycle (Met, SAM, and SAH) and of polyamine synthesis (spermidine, ornithine, and putrescine) (Figure 4a) in wild‐type and *dilp2‐3,5* mutant flies in the presence and absence of *Gnmt* (*Gnmt^Mi^* and *Gnmt^Mi^*,*dilp2‐3,5* double mutants). Two metabolites of the Met cycle, Met and SAH, were significantly increased in response to reduced IIS, and the level of SAH, but not Met, was dependent on the presence of Gnmt (Figure 4b). Interestingly, spermidine levels were significantly increased in *dilp2‐3,5* mutant flies compared to controls (Figure 4b), but TSP and glutathione pathway metabolites (cysteine, cystathionine, γ‐Glu‐Cys, and glutathione) were unchanged (Figure S4b). Furthermore, the IIS‐mediated increase in spermidine levels required *Gnmt* (Figure 4b). These findings suggest that reduced IIS activity can modulate methionine metabolism and polyamine synthesis, but not the TSP or glutathione, through Gnmt (Figure 4b). This supports our previous conclusion that the beneficial role of Gnmt in response to reduced IIS does not occur through the TSP or glutathione pathways, but rather through spermidine synthesis.

To confirm the role of Gnmt and spermidine in IIS‐mediated changes to Met metabolism, we treated wild‐type and *dilp2‐3,5* mutant flies with spermidine in the presence and absence of *Gnmt* (*Gnmt^Mi^* and *Gnmt^Mi^*,*dilp2‐3,5* double mutants) (1mM) and quantified associated metabolites (Figure 4b). Treatment with spermidine led to restricted changes in metabolism, changing only SAM and putrescine levels in *Gnmt^MI^* and *dilp2‐3,*5 mutant flies, respectively, and spermidine levels in wild‐type (*wDah*) and *dilp2‐3,5; gnmt* double mutant flies (Figure 4b). However, two‐way ANOVA revealed a significant interaction between spermidine treatment and genotype, *dilp2‐3,5* mutants showed no increase in spermidine levels, as these mutants maintain elevated spermidine levels, independently of spermidine supplementation (Figure 4b). Interestingly, the loss of elevated spermidine levels in *dilp2‐3,5* mutants in the absence of *Gnmt* was completely abrogated by treatment with spermidine (Figure 4b). Together, these results show that reduced IIS increases spermidine synthesis and that this increase requires Gnmt.

To determine the role of Gnmt in spermidine production and IIS mutant longevity, we assayed the lifespan of wild‐type, *Gnmt^Mi^* and *dilp2‐3,5* single mutant, and *Gnmt^Mi^*,*dilp2‐3,5* double mutant flies with or without spermidine supplementation. Spermidine increased the lifespan of wild‐type flies, but not of either *Gnmt^Mi^* or *dilp2‐3,5* mutants (Figure 4c), suggesting that the elevated spermidine level in these mutants was already sufficient to maximize their lifespan by this mechanism. Gnmt also appeared to exert its effects on lifespan through spermidine, because the reduction of the lifespan of the *dilp2‐3,5* flies by the *Gnmt^Mi^* mutant was completely abrogated when the double mutants were fed spermidine (Figure 4c). In accordance with our metabolic analysis (Figure 4b), the lifespan analysis indicated that spermidine regulates processes that play a major role in IIS‐mediated longevity.

### 2.6 Increased Gnmt activity and spermidine synthesis in IIS mutants induce expression of genes in the autophagy pathway

**Figure 5:**
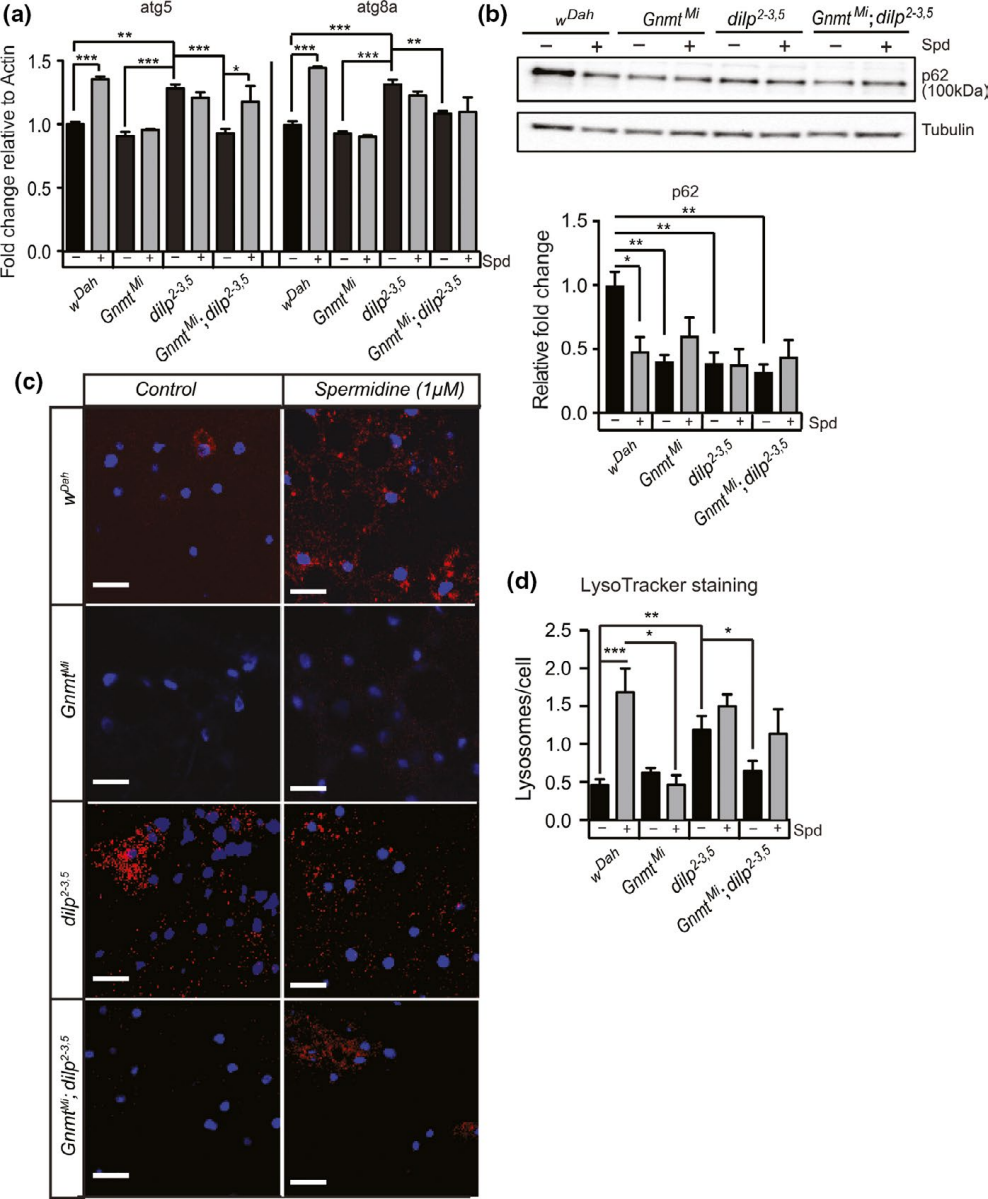
Enhanced Gnmt induces autophagy in the fat body of flies. (a) qRT–PCR quantification of *atg5* and *atg8a* transcript levels, (b) Western blot analysis of p62 protein levels, and (c) representative confocal microscope images of LysoTracker Red (red)‐stained vacuoles, indicative of autophagy, and DAPI‐stained nuclei (blue) in the fat body of *w^Dah^* control, *Gnmt^Mi^* mutant, *dilp2‐3,5* mutant, and *Gnmt^Mi^;dilp2‐3,5* double mutants under spermidine (1 mM) fed and control (SYA) fed conditions (d) Quantification of LysoTracker Red‐stained vacuoles per nucleus represented in (c). Chart shows mean and error bars represent *SEM* *w^Dah^* control (*n* = 11), *w^Dah^* Spd (*n* = 17), *Gnmt^Mi^* control (*n* = 9), *Gnmt^Mi^* Spd (*n* = 5), *dilp2‐3,5* control (*n* = 16), *dilp2‐3,5* Spd (*n* = 21), *Gnmt^Mi^;dilp2‐3,5* control (*n* = 14), *Gnmt^Mi^;dilp2‐3,5* Spd (*n* = 14). Significance determined by two‐way ANOVA and pairwise post hoc tests (*p*‐value *< .05, **< .01, ***< .001)

Reductions in IIS can induce autophagy, and this induction is essential for lifespan extension in IIS mutants in *C. elegans* (Meléndez et al., 2003). Spermidine can induce autophagy in yeast, worms, flies, and mice (Eisenberg et al., 2009). To determine whether Gnmt plays a role in autophagy induction in response to reduced IIS, we investigated expression of autophagy associated genes in the fat bodies of wild‐type (wDah), *Gnmt^Mi^* and *dilp2‐3,5* single mutants, and *Gnmt^Mi^*,*dilp2‐3,5* double mutants fed either spermidine (1mM) or control food. Expression of *atg5* and *atg8a* was induced in the fat bodies of *dilp2‐3,5* mutants, but not in *Gnmt^Mi^* or *Gnmt^Mi^*,*dilp2‐3,5* double mutant flies compared to controls (Figure 5a). Induction of both *atg5* and *atg8a* is thus directly, or indirectly, regulated through Gnmt, in response to reduced IIS. Furthermore, spermidine treatment led to induction of both *atg5* and *atg8a* in wild‐type flies suggesting, in agreement with previous studies (Eisenberg et al., 2009), that spermidine treatment induces autophagy, but it did so only in the presence of Gnmt (Figure 5a). Spermidine treatment did not increase the level of *atg5* and *atg8a* expression beyond that of control fed *dilp2‐3,5* (Figure 5a). However, in *dilp2‐3,5* mutants lacking *Gnmt*, spermidine treatment increased *atg5*, but not *atg8a*, expression (Figure 5a). Together, the induction of *atg5* and *8a* expression suggests that autophagy is induced in response to reduced IIS and that induction is dependent on *Gnmt.* Furthermore, in the case of *atg5*, spermidine treatment is sufficient to recapitulate the induction of autophagy seen in IIS mutants, even in the absence of *Gnmt.*

We then used Western blot analysis to quantify the level of p62, a substrate of autophagy. Increased levels of p62 suggest autophagy is blocked or slowed, while decreased levels suggest increased autophagy (Nagy, Varga, Kovács, Takáts, & Juhasz, 2015). The level of p62 was significantly decreased in the fat body of *dilp2‐3,5* mutants, *Gnmt^Mi^* mutants, and *Gnmt^Mi^*,*dilp2‐3,5* double mutant flies compared to controls (Figure 5b). Treatment with spermidine significantly reduced p62 levels in the fat body of wild‐type flies, but not in the fat body of *dilp2‐3,5* mutants, *Gnmt^Mi^* mutants, or *Gnmt^Mi^*,*dilp2‐3,5* double mutant flies, when compared to untreated control flies (Figure 5b). Thus, autophagy in the fat body is activated in response to reduced IIS or spermidine treatment. Surprisingly, p62 levels are also reduced in the fat body of *Gnmt^Mi^* flies and *Gnmt^Mi^*,*dilp2‐3,5* double mutant flies. This suggests that Gnmt may also play a role in autophagy activation in response to spermidine treatment.

To determine whether the responses to reduced IIS and spermidine treatment led to functional changes in autophagy, we quantified the level of autophagy using LysoTracker Red (LTR) in the fat bodies of control and *dilp^2‐3,5^* mutant flies in the presence and absence of Gnmt and with and without spermidine treatment. LTR accumulates inside acidic vesicles allowing direct assessment of autophagic status of a tissue. The fat body of *dilp^2‐3,5^* mutant flies showed significantly higher numbers of LTR‐stained vesicles per cell compared to controls flies, suggesting increased levels of autophagy (Figure 5b,c). This suggests reducing IIS can increase autophagy in the fat body. Importantly, the observed increase in autophagy in *dilp2‐3,5* mutants did not occur in the absence of *Gnmt,* and *Gnmt^Mi^* mutants show no changes in autophagic status compared to controls (Figure 5b,c)*.* This suggests that, either directly or indirectly, Gnmt modulates autophagy specifically in IIS mutant flies.

The fat body of wild‐type flies fed with spermidine (1mM) showed a significant increase in autophagy compared to untreated control flies, increasing to the level of untreated *dilp2‐3,5* mutants (Figure 5b,c). Spermidine treatment did not further enhance autophagy in *dilp2‐3,5* mutants (Figure 5b,c). Furthermore, supporting our analysis of *atg5 and atg8a* expression (Figure 5a), *Gnmt^MI^* flies did not increase the level of autophagy in response to spermidine treatment (Figure 5b,c). However, treatment of *dilp2‐3,5* mutants, in the absence of *Gnmt,* with spermidine increased autophagy activation, but not significantly above the level in controls (Figure 5b,c). Thus, *Gnmt* plays a role in modulating autophagy in response to reduced IIS, and spermidine may be a component within that response.

### 2.7 Evolutionarily conserved response of Gnmt in liver‐specific *IRS1* KO mice

**Figure 6:**
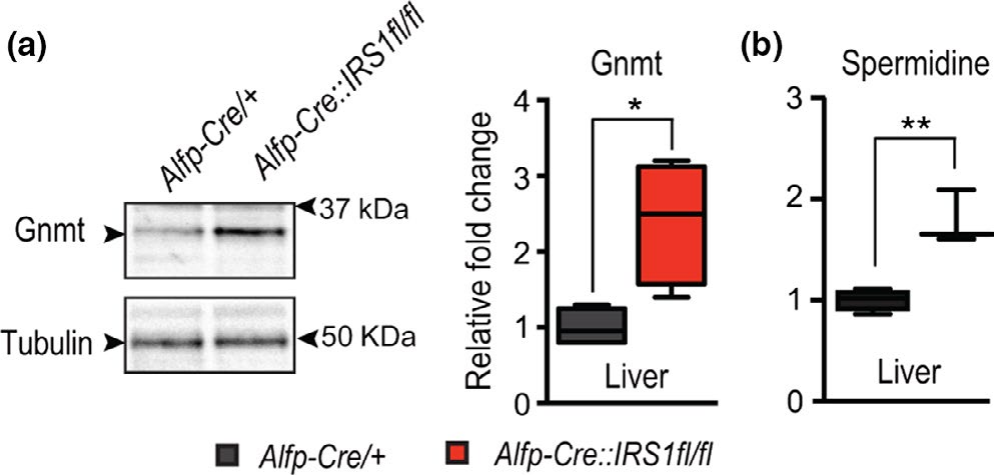
Evolutionarily conserved response of Gnmt in long‐lived mouse models. (a) Western blot quantification of Gnmt protein in the liver of liver‐specific *IRS1* KO mice (Alfp‐Cre::*IRS*1fl/fl) compared to control mice (3 months) siblings (*n* = 4, *p*‐value **< .01, ***< .001; Student's *t* test)

Gnmt is an evolutionarily conserved regulator of methionine metabolism, performing the same function in both flies and mice (Obata et al., 2014). To determine whether increased expression of Gnmt in response to reduced IIS is also conserved between mice and flies, we examined Gnmt levels in the functional equivalent of the fly fat body, the liver, of liver‐specific *IRS1* KO (Alfp‐Cre::*IRS*1fl/fl) mice (Essers et al., 2016). We confirmed by Western blot that, as in flies, reducing IIS activity resulted in significantly increased levels of *Gnmt* in the liver (Figure 6a). To determine whether the increased expression of Gnmt led to conserved metabolic changes, we then quantified the spermidine in the liver of liver‐specific *IRS1* knockout mice (Figure 6b). In agreement with our previous analysis in IIS mutant flies, liver‐specific loss of IRS1 increased levels of spermidine (Figure 6b). Together, these results suggest that the response of increasing Gnmt expression is a conserved response to reduced IIS activity and that this response may be mechanistically similar, acting through spermidine.

## 3 DISCUSSION

Finding modulators of aging through better understanding of the precise mechanistic responses to prolongevity perturbations is of vital importance to translating interventions from the laboratory bench to the clinic. Understanding how it all fits together and where to most effectively intervene are the current big issues. We have shown that, in response to reduced IIS, Met metabolism is altered, through the tissue‐specific transcriptional up‐regulation of the glycine N‐methyl transferase, Gnmt. The level of *Gnmt* was elevated in the fat body of long‐lived IIS mutant flies. This is in agreement with a recent proteomic study of an independent IIS mutant (Tain et al., 2017) which showed fat body‐specific increases in Gnmt, suggesting that increased Gnmt levels, and thus altered Met metabolism, may be a common feature of long‐lived IIS models. Recently, Gnmt expression was found to be elevated in long‐lived dietary restricted mice (Hahn et al., 2017), and increased Gnmt expression is a common feature of long‐lived Ames dwarf mice (Brown‐Borg, Borg, Meliska, & Bartke, 1996). Together, this suggests that modulation of Met metabolism, through Gnmt, may be common to long‐lived models.

We show that Gnmt is required for the longevity of IIS mutant flies and that fat body‐specific over‐expression of *Gnmt* is sufficient to extend the lifespan of otherwise wild‐type flies. Due to the central position of Gnmt within the Met cycle, Gnmt activity can influence the production of several metabolites. In *Drosophila*, loss of Gnmt has been reported to increase the levels of SAM and methionine and reduce the levels of SAH and sarcosine in male hemolymph (Obata et al., 2014). Our analysis of total metabolites in females (hemolymph and nonhemolymph) could not recapitulate those precise metabolic changes in *Gnmt^Mi^* mutants, suggesting they may be specific to the hemolymph or influenced by gender, diet, and fly strain. Gnmt activity also confers influence over the transsulfuration, glutathione production, and polyamine pathways (Luka et al., 2009; McIsaac et al., 2016). Each of the above pathways has been linked to aging (Ayyadevara et al., 2005; Eisenberg et al., 2009; Kabil et al., 2011). Our findings suggest that the role of Gnmt in IIS‐mediated longevity is independent of both TSP activity and glutathione production. Neither H_2_S production nor increased oxidative stress resistance outputs from the TSP and glutathione pathways, respectively, were altered in our long‐lived flies. Indeed, in response to reduced IIS, Met metabolism was altered through Gnmt activity, resulting in elevated levels of the polyamine spermidine. As spermidine synthesis is not within the normal enzymatic function of Gnmt, it remains possible that the increase in spermidine and other metabolites is indirect, or via changes in flux through the pathway. Autophagy was also activated in response to reduced IIS, possibly through Gnmt and altered spermidine levels. This response of autophagy to reduced IIS and its interaction with spermidine suggests that other regulators of autophagy may play a role. Furthermore, highlighting the importance of spermidine in IIS mutants, we show that the abolition of IIS mutant longevity in the absence of *Gnmt* can be entirely suppressed by feeding flies with the polyamine spermidine.

Polyamines are promiscuous polycations, and thus, one of their main features is to interact with negatively charged molecules, including DNA, RNA, and proteins. Declining levels of polyamines, including spermidine, occur with aging in humans (Vivó et al., 2001). Treatment with one polyamine, spermidine, can extend lifespan in multiple species (Eisenberg et al., 2016, 2009). Spermidine treatment can also maintain mitochondrial volume and prevent loss of mitochondrial respiration in aged mice (Eisenberg et al., 2009). We show that spermidine levels are increased in long‐lived IIS mutant flies. Interestingly, we have previously shown that the fat body of IIS mutant flies shows increased mitochondrial biogenesis and respiration (Tain et al., 2017). Furthermore, increased mitochondrial biogenesis and respiration were both necessary and sufficient for the longevity of IIS mutant flies (Tain et al., 2017). This suggests that increased mitochondrial biogenesis and respiration may be a common downstream response to altered Met metabolism. Eisenberg *et al.* suggest that the beneficial effects of spermidine treatment on mitochondrial homeostasis occur through increased mitophagy (Eisenberg et al., 2016). IIS mutant flies have increased spermidine levels and, through spermidine, have increased activation of autophagy. Increased autophagic activity may in turn increase mitophagy. Thus together, increased autophagy, including mitophagy, and increased mitochondrial biogenesis in coordination may preserve tissue homeostasis and underlie the longevity of IIS mutants.

Finally, we show that these responses to reduced IIS are evolutionarily conserved. Liver‐specific loss of IRS1 was sufficient to increase *Gnmt* expression, alter Met metabolism, and increase the level of spermidine in mice. In summary, our study highlights spermidine synthesis as a positive downstream regulator of IIS‐mediated longevity in flies. Furthermore, this response is evolutionarily conserved and therefore could offer insights into future prolongevity therapeutics.

## 4 EXPERIMENTAL PROCEDURES

### 4.1 *Drosophila* genetics and lifespan assay

Flies were maintained in a controlled temperature environment of 25°C, 65% humidity with a 12‐hr day–night light cycle, and fed standard sugar/yeast/agar (SYA) diet (Bass et al., 2007). Larval densities were controlled, and adults were once mated. Female flies were sorted under CO_2_ at 10 flies/vial. Lifespan analysis was performed using SYA food or SYA food containing 200μM of RU‐486, 1mM spermidine, or vehicle (EtOH) control food, and survival was scored every 2–3 days.

Food containing RU486 (200 μM) and/or spermidine (1 mM) was prepared every two weeks from a 100 mM (in ETOH) or a 1 M (in ddH2O) stock solution, respectively.

RU486, spermidine, or vehicle control supplementation commenced 48 hr posteclosion. Food vials were changed at 2‐ to 3‐day intervals. Lifespan data used to generate survival curves are summarized in Supporting Information Table S1. All flies were backcrossed for 10 generations into the white Dahomey (*w^Dah^*) background. The genotypes used were *w^Dah^*, *dilp2‐3,5* mutant (Grönke et al., 2010), *Gnmt^Mi04290^* mutant (Venken et al., 2011), and *Gnmt^Mi04290^* mutant in a *dilp2‐3,5* background (*Gnmt^Mi04290^*;*dilp2‐3,5*). For over‐expression analysis, *UAS‐Gnmt* (Obata & Miura, 2015) flies were crossed with *FB‐GAL4* and *pumpless‐GAL4* drivers for constitutive expression of *Gnmt* in the fat body, and with *S106GS‐GAL4* driver for adult‐onset expression.

### 4.2 Generation of tissue‐specific KO mice and maintenance

Liver‐specific knockout of *IRS1* mice was generated as described in Essers et al., 2016. Briefly, *IRS1^loxP/loxP^* mice were crossed with *Cre recombinase*‐expressing mice under the control of the mouse albumin enhancer and promoter and the mouse alpha‐fetoprotein enhancers. All mice were maintained at 22°C under a 12‐hr light/dark cycle with ad libitum access to normal chow [ssniff® R/M‐H phytoestrogen‐poor (9% fat, 34% protein, 57% carbohydrate) Spezialdiäten GmbH, Soest, Germany] and water. Mice were sacrificed at 3–4 months. Livers were dissected and snap‐frozen in liquid nitrogen.

### 4.3 Western blots

Total protein (30 μg) was loaded and separated on precast TGX gels (Any kD, Bio‐Rad) and transferred to nitrocellulose (0.45 μm) membranes (GE Healthcare). Membranes were incubated (1 hr) in blocking solution (5% nonfat milk in 0.05% TBST) at room temperature and then in primary antibody anti‐Gnmt (for detection of mouse Gnmt (1:1,000)(sc‐68871) or of fly anti‐Gnmt (1:1,000)) (a generous gift from Prof. Miura), anti‐p62 (1:5,000) (a generous gift from Prof. Juhász), and tubulin (1:10,000)(Cell Signaling Technologies) overnight at 4°C. Membranes were washed three times with TBST (0.05%) for 10 min. HRP‐conjugated anti‐mouse (1:10,000 dilution) or anti‐rabbit (1:10,000 dilution) secondary antibodies were incubated for 1 hr at room temperature. Blots were then washed three times with TBST (0.05%), and detection was performed with ECL Prime reagent (Amersham). Blots were imaged in a ChemiDoc^TM^ imaging station, and protein bands were quantified using Image Lab software (Bio‐Rad).

### 4.4 qRT–PCR

Total RNA was extracted using TRIzol (Invitrogen) to manufacturers’ guidelines and subsequently treated with DNase I (Ambion). cDNA was synthesized using SuperScript® VILO Master Mix (Invitrogen) cDNA Synthesis Kit, following the manufacturers’ protocol. Quantitative real‐time PCR (qRT–PCR) was performed using SYBR Green probes (Applied Biosystems) on a 7900HT Real‐Time PCR System (Applied Biosystems).

The primers used in the study are listed below:

**Table:** 

samdc	FP: ACGTGCTTAGCAATGTCAACTG RP: GCAACTGACCCAGGCATTTC
odc1	FP: GTGCAATGACGATCCAATGGT RP: CTCCGGCGAGACATCGAAG
rpl32	FP: ATATGCTAAGCTGTCGCACAAATGG RP: GATCCGTAACCGATGTTGGGCA

### 4.5 DDT and H_2_O_2_ assay

For DDT and H_2_O_2_ stress assay, 100 female flies (20 flies/vial) were initially fed either SYA food, RU‐486 (200 μM), or ethanol control food for 7 days. Flies were then transferred into fresh vials containing 0.03% DDT mixed in SYA food for xenobiotic stress assay and 1% agar food containing 5% v/v H_2_O_2_ for oxidative stress assay, respectively. The number of dead flies was counted four times a day and scored in an Excel sheet.

### 4.6 H_2_S measurement

For quantification of H_2_S production, 100 female flies were sorted into vials (20 flies/vial) and fed either SYA food, RU‐486 (200 μM), or ethanol control food. Flies were transferred into fresh vials every second day. H_2_S measurements were performed as previously described (Hine et al., 2015). Briefly, 20 aged (10 days) flies were chilled on ice and lysed in 300 μl lysis buffer (5× passive lysis buffer (Promega) made up to 1× lysis buffer with PBS containing cysteine (10 mM) and PLP (10 μM)). 10 μl of lysate was removed for later BCA protein quantification (Pierce) and normalization. Fly lysate was then transferred to a 96‐well plate, and individual wells were tightly covered with lead acetate paper and incubated at 37^oc^ (3 hr). H2S production was then assayed by densitometry analysis (ImageJ) of lead sulfide darkening and normalized to protein content.

### 4.7 Measurement of metabolites by LC‐MS/MS

For measurement of metabolites (Sigma), an Acquity UPLC^TM^ I‐Class System/Xevo^TM^ TQ‐S (Waters^TM^) with MassLynx was used. In brief, 25 snap‐frozen female flies were homogenized in 71 μl of 15 mg/ml dithiothreitol (DTT) and 429 μl 50% methanol (total volume 500 μl) and ~35 mg of frozen mouse tissues were homogenized in 1ml sample buffer (methanol:H_2_O:chloroform in 7:2:1 ratio). Homogenates were mixed for 5 min and centrifuged at 9,300 *g* at 4°C for 15 min. The supernatant was filtered using 0.2‐μm VWR centrifugal filters at maximum speed at 4°C for 5 min. The filtrate was then evaporated using SpeedVac for approx. 2 hr at 30°C. The pellet was reconstituted into 100 μl of running buffer (5mM ammonium formate, 0.15% formic acid aqueous solution, and 100 μg/ml DTT) and filtered again. Each metabolite was run in 1:10 and 1:100 dilutions to meet the standard run range. For absolute quantification of metabolites (Sigma) in positive ESI MRM (multireaction monitoring) mode, an Acquity UPLC^TM^ I‐Class System/Xevo^TM^ TQ‐S (Waters^TM^) with MassLynx and absolute quantification TargetLynx^TM^ (Waters^TM^) were used. With settings for capillary kv 1.5, desolvation temp. 550°C, desolvation gas flow 800 L/Hr, cone 150 L/Hr, and collision gas flow 0.15 ml/min, a Supelco^TM^ Discovery^TM^ HS F5‐3 Column from Sigma 3 µm × 2.1 mm × 100 mm was used at 25°C. Solvent A was 5 mM ammonium formate (Sigma) + 0.15% formic acid (Sigma) and B acetonitrile (VWR). A gradient from 100% A to 0% in 8 min at a flow rate of 0.35 ml/min and an equilibration step from 8.3 to 15 min were used. The following MRM transitions were used as quantifier (M + H^+^)^+^ for putrescine 89.10*–*30.14 *m/z*, l‐cysteine hydrochloride 121.92*–*75.93 *m/z*, ornithine 133.03–70.09 *m/z*, spermidine 146.14–72.16 *m/z*, l‐methionine 150.03–55.99 *m/z*, Cys‐Gly 178.85–75.93 *m/z*, l‐cystathionine 222.96–87.90 *m/z*, y‐Glu‐Cys 250.96–83.94 *m/z*, l‐glutathione reduced 308.02–75.92 *m/z*, SAH 385.10–136.38 *m/z*, and SAM 399.10–250.03 *m/z*. Compounds were dissolved in 5 mM ammonium formate + 0.15% formic acid + 100 µg/ml DTT. For all compounds, a calibration curve was calculated. Using concentrations from 5 to 75,000 ng/ml (prepared from stock solutions 100 µg/ml), correlation coefficient: *r* < 0.990; response type: external standard, the peak integrations were corrected manually, if necessary. Quality control standards of each standard were used during sample analysis and showed between 0.5% and 40% deviation, respectively. Blanks after the standards, quality control, and sample batch proved to be sufficient. Absolute value of metabolites was normalized to protein content. Relative fold change value was measured to determine the changes in amount of metabolites.

### 4.8 LysoTracker staining and quantification

For LysoTracker staining, fat bodies from female flies (10 days) were dissected in PBS and immediately stained with LysoTracker Red DND‐99 (Invitrogen) dye (1 μM in PBS) for 2 min. Tissues were then washed three times in PBS and mounted in mounting medium (VECTASHIELD H‐1200) containing DAPI. Images were taken using confocal microscope (Leica TCS SP5X) with 40x 1.25 oil objective. Laser power and optical settings were kept constant between images. Images were then analyzed using Imaris 8.0 software. The number of LysoTracker puncti and DAPI‐stained nuclei was quantified according to user manual guidelines and puncti/nucleus calculated. All image analysis and quantification were performed under blinded conditions.

### 4.9 Statistical analysis

Statistical analysis was performed using GraphPad Prism software 5.0f. Individual statistical tests are mentioned in appropriate figure legends. Lifespan assays and stress survival assays were recorded using Excel, and survival was analyzed using log‐rank test.

## AUTHOR CONTRIBUTIONS

LT conceived of the study, participated in its design and coordination, carried out most of the experiments, and drafted the manuscript. CJ participated in the coordination of the study and experimental work. TN generated mouse tissue. JF participated in experimental work. YH participated in experimental work. SG participated in the design and coordination of the study. LP conceived of the study, participated in its design and coordination, and drafted the manuscript.

## References

[acel13043-bib-0001] Afschar, S. , Toivonen, J. M. , Hoffmann, J. M. , Tain, L. S. , Wieser, D. , Finlayson, A. J. , … Partridge, L. (2016). Nuclear hormone receptor DHR96 mediates the resistance to xenobiotics but not the increased lifespan of insulin‐mutant *Drosophila* . Proceedings of the National Academy of Sciences, 113, 1321–1326. 10.1073/pnas.1515137113 PMC4747718 26787908

[acel13043-bib-0002] Armstrong, V. L. , Rakoczy, S. , Rojanathammanee, L. , & Brown‐Borg, H. M. (2014). Expression of DNA methyltransferases is influenced by growth hormone in the long‐living ames dwarf mouse in vivo and in vitro. Journals of Gerontology. Series A, Biological Sciences and Medical Sciences, 69, 923–933. 10.1093/gerona/glt133 PMC4111294 24201695

[acel13043-bib-0003] Ayyadevara, S. , Engle, M. R. , Singh, S. P. , Dandapat, A. , Lichti, C. F. , Benes, H. , … Zimniak, P. (2005). Lifespan and stress resistance of *Caenorhabditis elegans* are increased by expression of glutathione transferases capable of metabolizing the lipid peroxidation product 4‐hydroxynonenal. Aging Cell, 4, 257–271. 10.1111/j.1474-9726.2005.00168.x 16164425

[acel13043-bib-0004] Brown‐Borg, H. M. , Borg, K. E. , Meliska, C. J. , & Bartke, A. (1996). Dwarf mice and the ageing process. Nature, 384, 33 10.1038/384033a0 8900272

[acel13043-bib-0005] Das, R. , & Kanungo, M. S. (1982). Activity and modulation of ornithine decarboxylase and concentrations of polyamines in various tissues of rats as a function of age. Experimental Gerontology, 17, 95–103. 10.1016/0531-5565(82)90042-0 7106211

[acel13043-bib-0006] Dobson, A. J. , He, X. , Blanc, E. , Bolukbasi, E. , Feseha, Y. , Yang, M. , & Piper, M. D. W. (2018). Tissue‐specific transcriptome profiling of *Drosophila* reveals roles for GATA transcription factors in longevity by dietary restriction. NPJ Aging Mech Dis, 4, 5 10.1038/s41514-018-0024-4 29675265 PMC5904217

[acel13043-bib-0007] Eisenberg, T. , Abdellatif, M. , Schroeder, S. , Primessnig, U. , Stekovic, S. , Pendl, T. , … Madeo, F. (2016). Cardioprotection and lifespan extension by the natural polyamine spermidine. Nature Medicine, 22(12), 1428–1438. 10.1038/nm.4222 PMC5806691 27841876

[acel13043-bib-0008] Eisenberg, T. , Knauer, H. , Schauer, A. , Büttner, S. , Ruckenstuhl, C. , Carmona‐Gutierrez, D. , … Madeo, F. (2009). Induction of autophagy by spermidine promotes longevity. Nature Cell Biology, 11, 1305–1314. 10.1038/ncb1975 19801973

[acel13043-bib-0009] Essers, P. , Tain, L. S. , Nespital, T. , Goncalves, J. , Froehlich, J. , & Partridge, L. (2016). Reduced insulin/insulin‐like growth factor signaling decreases translation in. Nature Publishing Group, 1–9. 10.1038/srep30290 PMC4959029 27452396

[acel13043-bib-0010] Fontana, L. , Partridge, L. , & Longo, V. D. (2010). Extending healthy life span‐from yeast to humans. Science, 328, 321–326. 10.1126/science.1172539 20395504 PMC3607354

[acel13043-bib-0011] Grandison, R. C. , Piper, M. D. W. , & Partridge, L. (2009). Amino‐acid imbalance explains extension of lifespan by dietary restriction in *Drosophila* . Nature, 462, 1061–1064. 10.1038/nature08619 19956092 PMC2798000

[acel13043-bib-0012] Grönke, S. , Clarke, D.‐F. , Broughton, S. , Andrews, T. D. , & Partridge, L. (2010). Molecular evolution and functional characterization of *Drosophila* insulin‐like peptides. PLoS Genetics, 6, e1000857 10.1371/journal.pgen.1000857 20195512 PMC2829060

[acel13043-bib-0020] Grönke, S. , Beller, M. , Fellert, S. , Ramakrishnan, H. , Jäckle, H. , & Kühnlein, R. P. (2003). Control of Fat Storage by a Drosophila PAT Domain Protein. Current Biology, 13(7), 603–606. 10.1016/S0960-9822(03)00175-1 12676093

[acel13043-bib-0013] Hahn, O. , Grönke, S. , Stubbs, T. M. , Ficz, G. , Hendrich, O. , Krueger, F. , … Partridge, L. (2017). Dietary restriction protects from age‐associated DNA methylation and induces epigenetic reprogramming of lipid metabolism. Genome Biology, 18, 56 10.1186/s13059-017-1187-1 28351387 PMC5370449

[acel13043-bib-0014] Hine, C. , Harputlugil, E. , Zhang, Y. , Ruckenstuhl, C. , Lee, B. C. , Brace, L. , … Mitchell, J. R. (2015). Endogenous hydrogen sulfide production is essential for dietary restriction benefits. Cell, 160, 132–144. 10.1016/j.cell.2014.11.048 25542313 PMC4297538

[acel13043-bib-0015] Jiang, H. , Patel, P. H. , Kohlmaier, A. , Grenley, M. O. , McEwen, D. G. , & Edgar, B. A. (2009). Cytokine/Jak/Stat signaling mediates regeneration and homeostasis in the *Drosophila* midgut. Cell, 137, 1343–1355. 10.1016/j.cell.2009.05.014 19563763 PMC2753793

[acel13043-bib-0016] Johnson, S. C. , Dong, X. , Vijg, J. , & Suh, Y. (2015). Genetic evidence for common pathways in human age‐related diseases. Aging Cell, 14, 809–817. 10.1111/acel.12362 26077337 PMC4568968

[acel13043-bib-0017] Kabil, H. , Kabil, O. , Banerjee, R. , Harshman, L. G. , & Pletcher, S. D. (2011). Increased transsulfuration mediates longevity and dietary restriction in *Drosophila* . Proceedings of the National Academy of Sciences, 108, 16831–16836. 10.1073/pnas.1102008108 PMC3189063 21930912

[acel13043-bib-0018] Kabil, O. , & Banerjee, R. (2010). Redox biochemistry of hydrogen sulfide. Journal of Biological Chemistry, 285, 21903–21907. 10.1074/jbc.R110.128363 20448039 PMC2903356

[acel13043-bib-0019] Kenyon, C. J. (2010). The genetics of ageing. Nature, 464, 504–512. 10.1038/nature08980 20336132

[acel13043-bib-0021] Longo, V. D. , Antebi, A. , Bartke, A. , Barzilai, N. , Brown‐Borg, H. M. , Caruso, C. , … Fontana, L. (2015). Interventions to Slow aging in humans: Are we ready? Aging Cell, 14, 497–510. 10.1111/acel.12338 25902704 PMC4531065

[acel13043-bib-0022] Lu, S. C. , & Mato, J. M. (2012). S‐adenosylmethionine in liver health, injury, and cancer. Physiological Reviews, 92, 1515–1542. 23073625 10.1152/physrev.00047.2011 PMC3698976

[acel13043-bib-0023] Luka, Z. , Mudd, S. H. , & Wagner, C. (2009). Glycine N‐methyltransferase and regulation of S‐adenosylmethionine levels. Journal of Biological Chemistry, 284, 22507–22511. 19483083 10.1074/jbc.R109.019273 PMC2755656

[acel13043-bib-0024] McIsaac, R. S. , Lewis, K. N. , Gibney, P. A. , & Buffenstein, R. (2016). From yeast to human: Exploring the comparative biology of methionine restriction in extending eukaryotic life span. Annals of the New York Academy of Sciences, 1363, 155–170. 10.1111/nyas.13032 26995762

[acel13043-bib-0025] Meléndez, A. , Tallóczy, Z. , Seaman, M. , Eskelinen, E.‐L. , Hall, D. H. , & Levine, B. (2003). Autophagy genes are essential for dauer development and life‐span extension in *C. elegans* . Science, 301, 1387–1391. 10.1126/science.1087782 12958363

[acel13043-bib-0026] Miller, D. L. , & Roth, M. B. (2007). Hydrogen sulfide increases thermotolerance and lifespan in *Caenorhabditis elegans* . Proceedings of the National Academy of Sciences, 104, 20618–20622. 10.1073/pnas.0710191104 PMC2154480 18077331

[acel13043-bib-0027] Miller, R. A. , Buehner, G. , Chang, Y. , Harper, J. M. , Sigler, R. , & Smith‐Wheelock, M. (2005). Methionine‐deficient diet extends mouse lifespan, slows immune and lens aging, alters glucose, T4, IGF‐I and insulin levels, and increases hepatocyte MIF levels and stress resistance. Aging Cell, 4, 119–125. 10.1111/j.1474-9726.2005.00152.x 15924568 PMC7159399

[acel13043-bib-0028] Minois, N. , Carmona‐Gutierrez, D. , & Madeo, F. (2011). Polyamines in aging and disease. Aging, 3, 716–732. 10.18632/aging.100361 21869457 PMC3184975

[acel13043-bib-0029] Murphy, C. T. , McCarroll, S. A. , Bargmann, C. I. , Fraser, A. , Kamath, R. S. , Ahringer, J. , … Kenyon, C. (2003). Genes that act downstream of DAF‐16 to influence the lifespan of *Caenorhabditis elegans* . Nature, 424, 277–283. 10.1038/nature01789 12845331

[acel13043-bib-0030] Nagy, P. , Varga, Á. , Kovács, A. L. , Takáts, S. , & Juhasz, G. (2015). How and why to study autophagy in *Drosophila*: It’s more than just a garbage chute. Methods, 151–161. 10.1016/j.ymeth.2014.11.016 25481477 PMC4358840

[acel13043-bib-0031] Narayan, V. , Ly, T. , Pourkarimi, E. , Murillo, A. B. , Gartner, A. , Lamond, A. I. , & Kenyon, C. (2016). Deep proteome analysis identifies age‐related processes in *C. elegans* . Cell Systems, 3, 144–159. 27453442 10.1016/j.cels.2016.06.011 PMC5003814

[acel13043-bib-0032] Obata, F. , Kuranaga, E. , Tomioka, K. , Ming, M. , Takeishi, A. , Chen, C.‐H. , … Miura, M. (2014). Necrosis‐driven systemic immune response alters SAM metabolism through the FOXO‐GNMT axis. Cell Reports, 7, 821–833. 10.1016/j.celrep.2014.03.046 24746817

[acel13043-bib-0033] Obata, F. , & Miura, M. (2015). Enhancing S‐adenosyl‐methionine catabolism extends *Drosophila* lifespan. Nature Communications, 6, 8332 10.1038/ncomms9332 PMC4595730 26383889

[acel13043-bib-0034] Osterwalder, T. , Yoon, K. S. , White, B. H. , & Keshishian, H. (2001). A conditional tissue‐specific transgene expression system using inducible GAL4. Proceedings of the National Academy of Sciences, 98, 12596–12601. 10.1073/pnas.221303298 PMC60099 11675495

[acel13043-bib-0035] Page, M. M. , Schuster, E. F. , Mudaliar, M. , Herzyk, P. , Withers, D. J. , & Selman, C. (2018). Common and unique transcriptional responses to dietary restriction and loss of insulin receptor substrate 1 (IRS1) in mice. Aging, 10, 1027–1052. 10.18632/aging.101446 29779018 PMC5990393

[acel13043-bib-0036] Partridge, L. , Deelen, J. , & Slagboom, P. E. (2018). Facing up to the global challenges of ageing. Nature, 561(7721), 45–56. 10.1038/s41586-018-0457-8 30185958

[acel13043-bib-0037] Pegg, A. E. (2009). Mammalian polyamine metabolism and function. IUBMB Life, 61, 880–894. 10.1002/iub.230 19603518 PMC2753421

[acel13043-bib-0039] Slack, C. , Giannakou, M. E. , Foley, A. , Goss, M. , & Partridge, L. (2011). dFOXO‐independent effects of reduced insulin‐like signaling in *Drosophila* . Aging Cell, 10, 735–748. 21443682 10.1111/j.1474-9726.2011.00707.x PMC3193374

[acel13043-bib-0040] Stout, G. J. , Stigter, E. C. A. , Essers, P. B. , Mulder, K. W. , Kolkman, A. , Snijders, D. S. , … Brenkman, A. B. (2013). Insulin/IGF‐1‐mediated longevity is marked by reduced protein metabolism. Molecular Systems Biology, 9, 679–679. 10.1038/msb.2013.35 23820781 PMC3734508

[acel13043-bib-0041] Tain, L. S. , Sehlke, R. , Jain, C. , Chokkalingam, M. , Nagaraj, N. , Essers, P. , … Partridge, L. (2017). A proteomic atlas of insulin signalling reveals tissue‐specific mechanisms of longevity assurance. Molecular Systems Biology, 13, 939 10.15252/msb.20177663 28916541 PMC5615923

[acel13043-bib-0042] Teleman, A. A. , Hietakangas, V. , Sayadian, A. C. , & Cohen, S. M. (2008). Nutritional control of protein biosynthetic capacity by insulin via Myc in *Drosophila* . Cell Metabolism, 7, 21–32. 10.1016/j.cmet.2007.11.010 18177722

[acel13043-bib-0043] Uhlén, M. , Fagerberg, L. , Hallström, B. M. , Lindskog, C. , Oksvold, P. , Mardinoglu, A. , … Asplund, A. , et al. (2015). Proteomics. Tissue‐based map of the human proteome. Science, 347, 1260419. 25613900 10.1126/science.1260419

[acel13043-bib-0044] Vivó, M. , de Vera, N. , Cortés, R. , Mengod, G. , Camón, L. , & Martínez, E. (2001). Polyamines in the basal ganglia of human brain. Influence of aging and degenerative movement disorders. Neuroscience Letters, 304, 107–111. 10.1016/S0304-3940(01)01776-1 11335066

[acel13043-bib-0045] Zinke, I. , Kirchner, C. , Chao, L. C. , Tetzlaff, M. T. , & Pankratz, M. J. (1999). Suppression of food intake and growth by amino acids in *Drosophila*: The role of pumpless, a fat body expressed gene with homology to vertebrate glycine cleavage system. Development, 126, 5275–5284. 10556053 10.1242/dev.126.23.5275

