# Supplementary figures and images for: Longevity in response to lowered insulin signaling requires glycine N‐methyltransferase‐dependent spermidine production
Source: Aging Cell. 2019 Nov 13;19(1):e13043. doi: 10.1111/acel.13043 (PMC6974722; doi:10.1111/acel.13043)

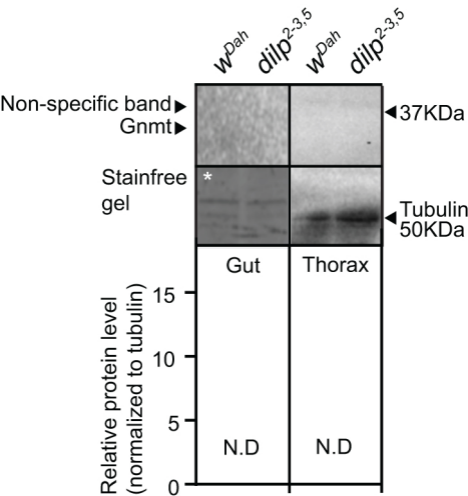

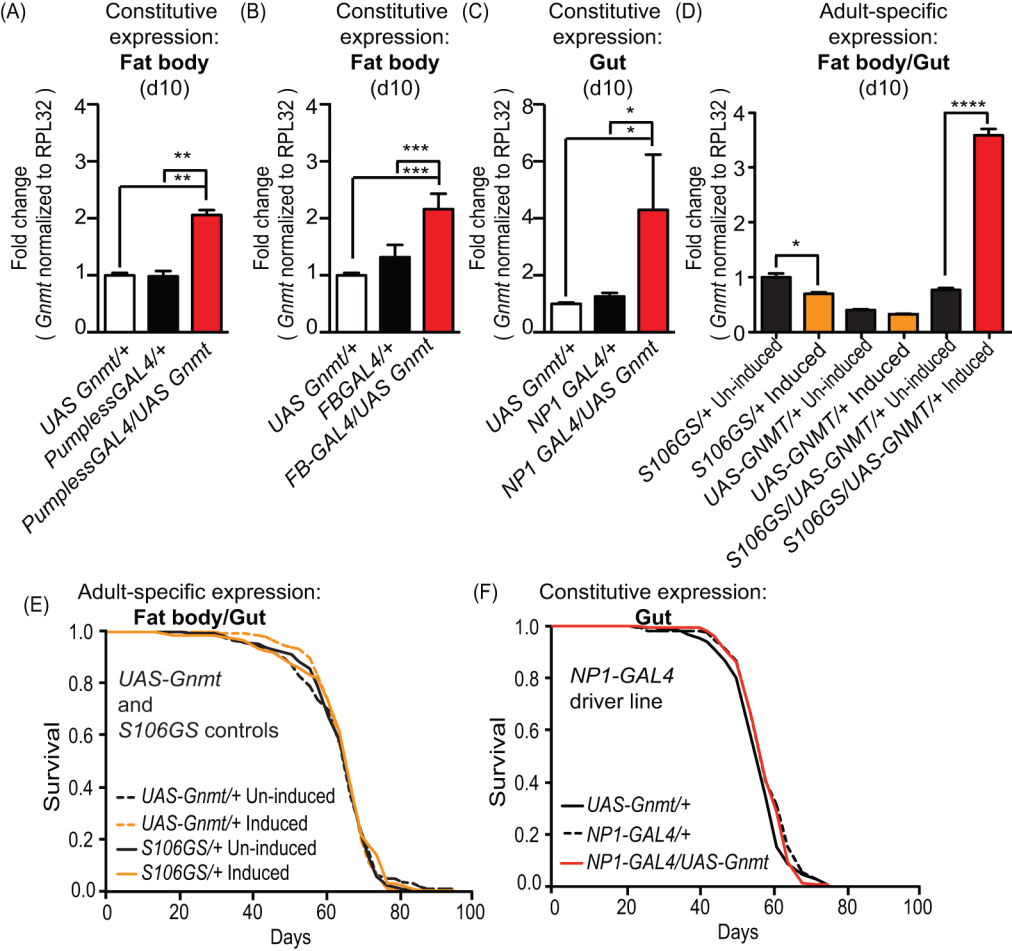

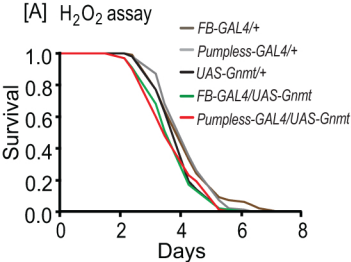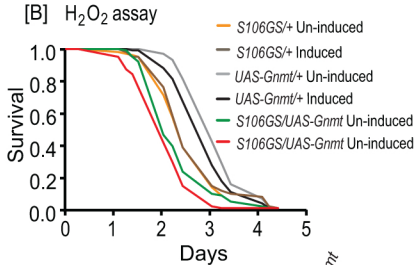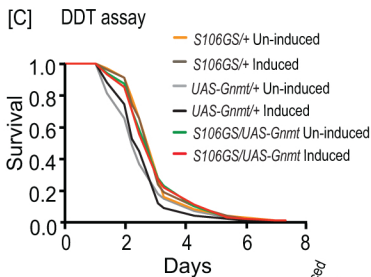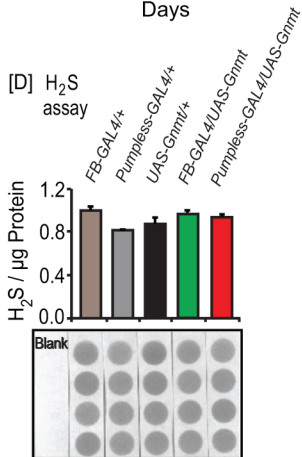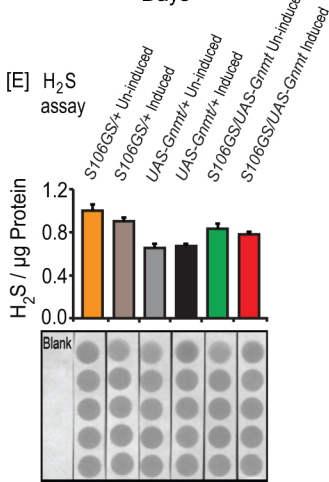

A

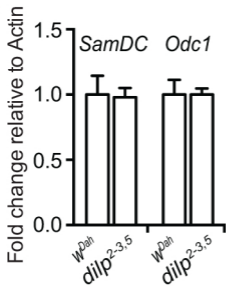

B

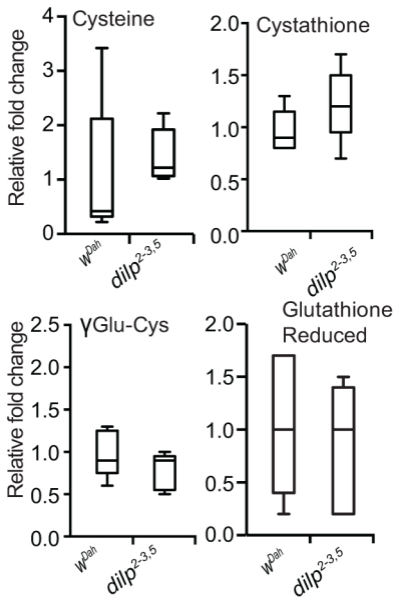

Supplement: Supplementary file 1 [file ACEL-19-e13043-s001.pdf]
